# Translation and validation of the polish version of the self-reported postural awareness scale in an adult sample

**DOI:** 10.3389/fpsyg.2025.1554594

**Published:** 2025-06-04

**Authors:** Agnieszka Jankowicz-Szymańska, Katarzyna Wódka, Aneta Grochowska, Anna Stefanowicz-Kocoł, Mirela-Ioana Bilc, Dennis Anheyer, Urszula Kozioł, Holger Cramer, Adam Sagan

**Affiliations:** ^1^Faculty of Medicine and Health Sciences, University of Applied Sciences in Tarnow, Tarnow, Poland; ^2^Institute of General Practice and Interprofessional Care, University Hospital Tübingen, Tübingen, Germany; ^3^Department of Market Analysis and Marketing Research, Faculty of Management, Cracow University of Economics, Cracow, Poland

**Keywords:** mindfulness, body posture, body sensations, perceived stress, body awareness

## Abstract

**Introduction:**

Body awareness has gained increasing attention in research as a crucial link between psychological and somatic processes, offering tangible benefits for physical health and well-being. This study aimed to validate and culturally adapt the Polish version of the Postural Awareness Scale (PAS) in adults aged 20–70.

**Methods:**

The relationships between the two PAS subscales: Ease/Familiarity with Postural Awareness and Need for Attention Regulation with Postural Awareness, and chronic stress levels (measured by the Perceived Stress Scale, PSS-10), as well as gender, age, and family status (individuals in permanent relationships versus singles), were explored. The factor structure was tested by exploratory and confirmatory factor analysis.

**Results and discussion:**

A total of 333 healthy participants (mean age: 36.74 ± 19.7 years; 76% female) completed the study. Cultural adaptation of the PAS required the removal of one item, resulting in an 11-item Polish version with strong internal consistency (Crohnbach’s α: 0.80–0.82) and psychometric properties comparable to the original German version. Multi-group analyses confirmed metric equivalence of the scale across age, gender, and family status. A negative correlation was observed between PAS scores and perceived stress (PSS-10), while no significant associations were found with gender or family status. Older participants exhibited higher scores on the Ease/Familiarity with Postural Awareness subscale. These findings suggest that the Polish version of the PAS is a reliable and valid tool for assessing postural awareness in diverse adult populations, with potential applications in research and clinical practice.

## Introduction

In recent years, we have witnessed the dynamic development of neuroscience, including cognitive neurophysiology (Mehling et al., 2011). Historically, a clear division between the body and mind has dominated biomedical fields such as medicine, physiotherapy, and nursing. However, increasing attention is now being directed toward body awareness, defined as the ability to perceive information originating from the body.^1^ Body awareness is a complex construct that is inherently challenging to assess, with no universally established standards.

Both insufficient and excessive body awareness can have adverse consequences. Limited body awareness may hinder learning new motor skills and negatively affect posture. In contrast, excessive sensitivity to bodily signals may lead to anxiety or hypochondria, referred to as maladaptive body awareness (Mehling et al., 2009). Conversely, adaptive body awareness is associated with improved pain management, facilitation of motor learning, and enhanced well-being and quality of life (Anderson, 2006).

A growing body of evidence highlights the interplay between bodily and emotional processes, suggesting that interoceptive and proprioceptive information can directly influence mood (Samain-Aupic et al., 2019). Research has also explored the reciprocal relationship between depression and physical patterns, such as gait (Lemke et al., 2000) and posture quality (Michalak et al., 2014). However, the empirical investigation of associations between body awareness and postural quality remains underexplored. Such studies are crucial for advancing knowledge in postural deformities’ treatment and improving interventions in related areas. Central to this endeavor is the availability of reliable and valid tools for assessing body awareness.

Existing tools for body awareness assessment, such as the Body Awareness Questionnaire (Shields et al., 1989), the Body Perception Questionnaire (BPQ) (Cabrera et al., 2018), and the Multidimensional Assessment of Interoceptive Awareness (MAIA) (Mehling et al., 2018), primarily focus on interoceptive sensations. For example, the Body Awareness Questionnaire measures sensitivity to body cycles, rhythms, and physiological changes. In turn, BPQ focuses on sensations from the organs of the neck, chest, and abdomen innervated by the autonomic nervous system. Although MAIA assesses multiple dimensions of body awareness, it does not specifically address aspects related to postural quality.

For further research on the connections between body awareness and body positioning in static and motion, it is necessary to prepare reliable measurement tools that can be used in different languages and cultures. Cultural adaptation of questionnaires enables a more nuanced understanding of the phenomena being studied. The target language version must be conceptually consistent with the original language version (Cruchinho et al., 2024). The lack of equivalence makes it impossible to compare the results of research conducted in groups differing in language and country (Beaton et al., 2000).

Given these gaps, the Postural Awareness Scale (PAS) (Cramer et al., 2018) was selected for adaptation and validation in Polish. The PAS was identified as particularly promising for investigating the relationship between body structure and proprioceptive sensations, based on expert consensus among the authors, including two physiotherapists specializing in posture correction. Additionally, as body awareness therapies have been shown to reduce stress and anxiety in populations such as pregnant women (Yüce et al., 2024), university students (Norheim et al., 2018), and patients with chronic psychosomatic symptoms (Landsman-Dijkstra et al., 2004), this study also examined correlations between postural awareness and perceived stress. The PSS-10 scale was selected because it had already been used in studies on the German population (a significant correlation between PSS-10 and PAS total R = −0.29, PAS factor 1 R = −0.24, and PAS factor 2 R = −0.23 was demonstrated (Cramer et al., 2018). Also, in the process of validating the English version of the PAS scale, a significant relationship was noted between body awareness and perceived stress in the entire study group and among mindfulness practitioners (Colgan et al., 2021).

## Materials and methods

### Postural awareness scale

The Postural Awareness Scale (PAS) was developed to assess self-awareness of body posture in adults (Cramer et al., 2018). The initial version of the scale included 42 items, which were subsequently reduced to 13 items. After removing an additional item, the final version comprises 12 items. The PAS evaluates two dimensions of postural awareness: Ease/Familiarity with Postural Awareness (items 1–5 and 12) and Need for Attention Regulation with Postural Awareness (items 6–11), which reflects an opposite tendency.

The PAS employs a 7-point Likert scale, ranging from 1 (“Not like me at all”) to 4 (“Neutral”) to 7 (“Completely like me”). To ensure consistent interpretation, six items are reverse-scored so that higher scores consistently indicate greater postural awareness. The PAS generates scores for each subscale as well as a total score. The maximum possible total score is 84, with a maximum of 42 points per subscale.

The original version of the PAS, developed in Germany, was validated in a sample of 512 adults with chronic pain (mean age: 50 ± 11 years). The scale demonstrated strong psychometric properties, including high internal consistency. Greater postural awareness was found to be associated with lower chronic pain intensity. Additionally, a 10-week multimodal mind–body program significantly increased postural awareness and reduced pain (Cramer et al., 2018).

The PAS has been translated and validated in several languages, including English (Colgan et al., 2021), Italian (Topino et al., 2020), Turkish (Dursun and Önen, 2024), and French (Da Costa Silva et al., 2022).

When adapting the PAS into Polish, the authors tried to follow the methodology presented by Cramer et al. (2018), who designed a scale to assess self-assessment of awareness in adults with chronic musculoskeletal pain. The original authors confirmed the validity and reliability of the tool. Construct validity was established using exploratory factor analysis, while internal consistency was confirmed using Cronbach’s alpha coefficient. Convergent validity should be assessed by calculating Pearson correlations between PAS scores and other validated tools measuring body awareness, body image, and mindfulness. However, since no other validated Polish scale was found for convergent validation, PAS scores were correlated with the level of stress expressed in the PSS-10 scale (the relationships between body awareness and stress levels were proven by, e.g., Cramer et al. (2018)).

### Translation and validation procedure

**Figure 1:**
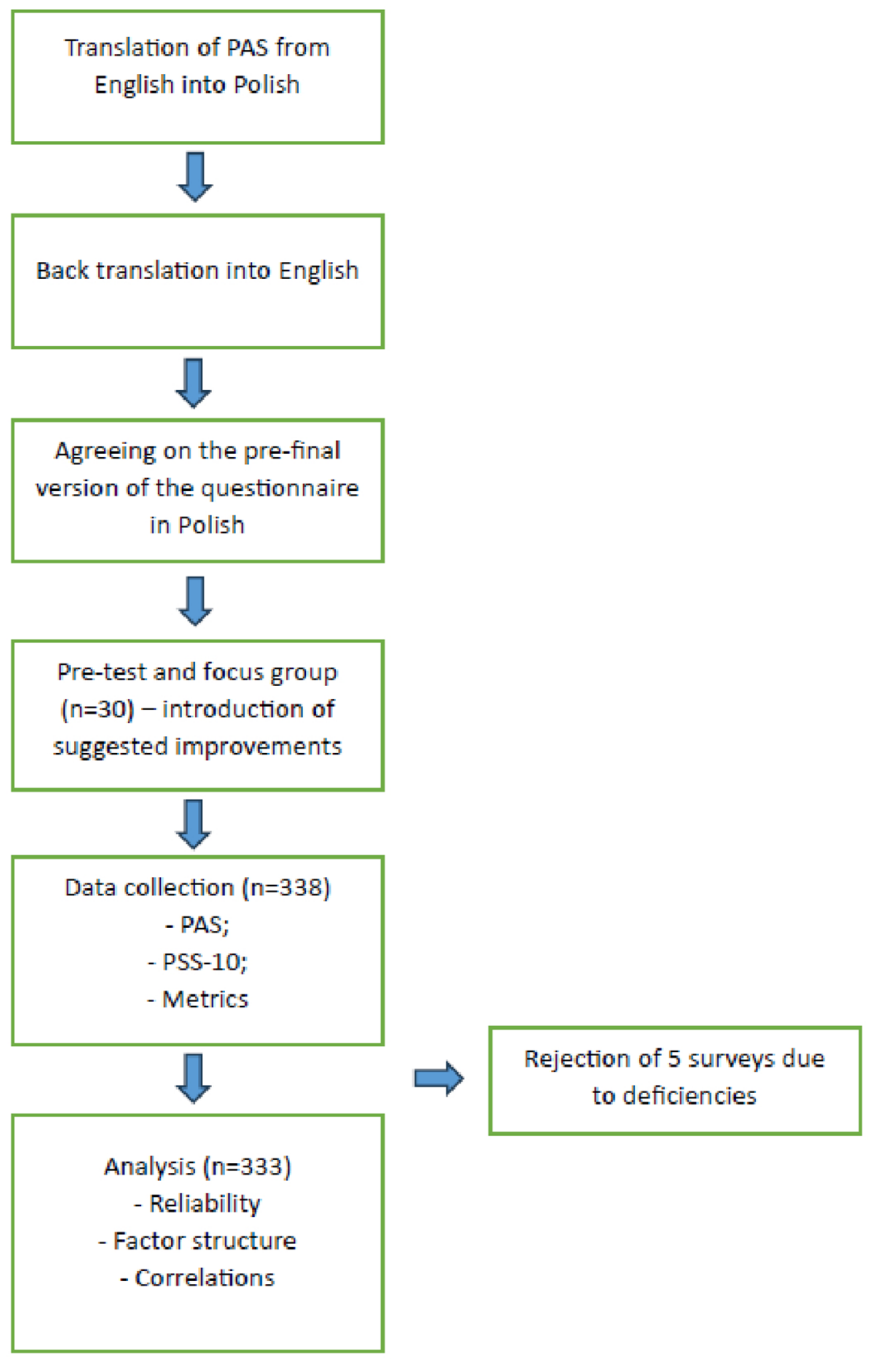
Procedure of the translation and validation process. PAS, postural awareness scale; PSS-10, perceived stress scale 10-item version.

The process of translating and validating the scale is outlined in Figure 1. The validated English version of the PAS was used for translation into Polish. The aim of the cultural validation was not to demonstrate cultural differences but to achieve cross-cultural equivalence in the original and polish language versions of the questionnaire (Huang and Wong, 2014). The involvement of bilingual people in the team preparing the forward and back translation, cooperation with the authors of the original version of the questionnaire and pilot studies in the target group have been implemented to ensure that all semantic and conceptual discrepancies are resolved.

The initial translation of the scale into Polish was performed independently by a physiotherapist and a nurse with advanced professional English proficiency. The inclusion of a nurse was intended to incorporate the perspective of a medical professional familiar with the topic while avoiding the use of technical physiotherapy-specific vocabulary that might not be easily understood by respondents. The primary goal was to produce a faithful translation, introducing modifications only where required by linguistic and cultural specificity. Two additional individuals (a physiotherapist and an English linguist) independently translated the Polish version back into English.

All four translators, along with a psychologist and personal development trainer, met in person to finalize the pre-final Polish version of the scale. Although the experts were not anonymous, the group adhered to the principles of the Delphi method (Landeta, 2006). All participants had equal standing in the discussions and were required to justify differing opinions. Arguments were exchanged until the group reached a consensus. The opinions of all members were taken into account to determine the final wording of each item.

In cases of uncertainty, the team consulted two additional English philologists and two psychologists with extensive professional experience. Special attention was given to the grammar, syntax, clarity, and logical coherence of each item. The names of both subscales were also carefully translated to ensure linguistic and conceptual alignment with the original version.

### Pre-test and focus group

Following the completion of the expert review, a pilot test was conducted to gather feedback on the Polish version of the PAS. Thirty participants (19 women and 11 men) aged between 20 and 25 years (mean age = 22.03 ± 1.03) were invited to complete the questionnaire. All participants were volunteers who provided informed consent and were informed about the purpose of the study. They were also told they could withdraw from the study at any time without providing any reason.

After completing the questionnaire, participants were asked to provide feedback on the following aspects: the clarity of the instructions in the header, the ease of understanding the wording of the items, the acceptability of the time required to complete the questionnaire, and any other difficulties encountered. Comments that were agreed upon by at least 15% of the participants were considered for revision. The proposed version of the PAS was well-received overall, with no feedback related to the clarity of the header or items, nor any difficulties in understanding the language (e.g., use of technical terms). The time required to complete the questionnaire was deemed acceptable.

### Perceived stress scale

The PSS-10 is a 10-item scale designed to assess the subjective stress level experienced in everyday situations over the past month. This tool is easy to administer and has demonstrated proven reliability and validity. The PSS-10 is commonly used to assess relationships between stress, behavioral disorders, and coping strategies in various conditions, including chronic illnesses (Bolkan Günaydın et al., 2022; Wieckiewicz et al., 2022; Blaettler et al., 2022; Katus et al., 2022; Boer et al., 2018). The scale uses a 5-point Likert scale (ranging from 0 to 4), with some items having reversed scoring. Higher scores indicate a higher level of perceived stress. The overall score is interpreted into a sten scale, which allows to define the stress level as low, moderate, or high.

### Validation procedure

Individuals aged 20 to 70 were invited to participate in the study. The invitation was posted electronically on the university’s Facebook page, where the study authors are employed. The invitation included instructions for completing the questionnaire, as well as information about exclusion criteria. Participants were informed that they should not complete the questionnaire if they met any of the following conditions:

- They are students of a medical or related field of study
- They work in the medical or healthcare professions
- They are experiencing severe pain or illness (e.g., fever)
- They have a disability
- They have suffered a musculoskeletal injury in the past 3 months
- They have a history of depression, eating disorders, or other mental health conditions

Data collection was conducted over a period of 8 weeks. The questionnaire was used in the form of an online Google form. Recruitment for the study was carried out in a way convenient for the authors, using social media and a nonprobability sampling technique where existing study participants were asked to invite their acquaintances to complete the questionnaire. No compensation for participation was provided. A total of 338 participants completed the survey. However, five responses were excluded from the analysis due to incomplete questionnaires (e.g., missing items from the PAS or PSS-10, or respondents leaving the questionnaire unfinished). The final analysis included data from 333 participants, meeting the minimum required sample size (10 times the number of items) as recommended by Terwee et al. (2007). Sociodemographic data for the study sample are presented in Table 1.

**Table 1:** Sociodemographic and clinical characteristics of the respondent group.

Stress level (% in row)	Low stress *n* = 46 (13.81%)	Moderate stress *n* = 82 (24.63%)	High stress *n* = 205 (61.56%)	Total *n* = 333 (100.00%)
Age in years (mean±SD)	38.76 ± 18.76	42.67 ± 21.88	33.93 ± 18.98	36.74 ± 19.96
Age group (% in column)				
18–35 years	22 (47.83%)	38 (46.34%)	133 (64.88%)	193 (57.96%)
36–60 years	16 (34.78%)	16 (19.51%)	40 (19.51%)	72 (21.62%)
>60 years	8 (17.39%)	27 (32.93%)	31 (15.12%)	66 (19.82%)
Prefer not to say	0 (0.00%)	1 (1.22%)	1 (0.49%)	2 (0.60%)
Gender, *n* (% in column)				
Female	26 (56.52%)	63 (76.83%)	164 (80.00%)	253 (75.98%)
Male	20 (43.48%)	18 (21.95%)	38 (18.54%)	76 (22.82%)
Prefer not to say	0 (0.00%)	1 (1.22%)	3 (1.46%)	4 (1.20%)
Family status, *n* (% in column)				
Stable relationship	32 (69.57%)	45 (54.88%)	109 (53.17%)	186 (55.86%)
Single, divorced, widower	13 (28.26%)	35 (42.68%)	95 (46.34%)	143 (42.94%)
Prefer not to say	1 (2.17%)	2 (2.44%)	1 (0.49%)	4 (1.20%)
Education, *n* (% in column)				
Higher education	25 (54.35%)	28 (34.15%)	69 (33.66%)	122 (36.64%)
Secondary education	19 (41.30%)	46 (56.10%)	126 (61.46%)	191 (57.36%)
Vocational education	2 (4.35%)	6 (7.32%)	8 (3.90%)	16 (4.80%)
Prefer not to say	0 (0.00%)	2 (2.44%)	2 (0.98%)	4 (1.20%)
Employment, *n* (% in column)				
Full-time	23 (50.00%)	20 (24.39%)	55 (26.83%)	98 (29.43%)
Part-time	1 (2.17%)	7 (8.54%)	8 (3.90%)	16 (4.80%)
Unemployed	0 (0.00%)	3 (3.66%)	5 (2.44%)	8 (2.40%)
Pension	7 (15.22%)	26 (31.71%)	36 (17.56%)	69 (20.72%)
Still learning	15 (32.61%)	25 (30.49%)	100 (48.78%)	140 (42.04%)
Prefer not to say	0 (0.00%)	1 (1.22%)	1 (0.49%)	2 (0.60%)
Chronic pain				
Yes, *n* (% in row)	7 (15.22%)	21 (25.61%)	65 (31.71%)	95 (28.53%)
Intensity (mean±SD)^1^	4.85 ± 2.85	5.37 ± 1.52	5.41 ± 2.11	5.36 ± 2.03
Duration in months (mean ± SD)^1^	42.42 ± 91.59	28.26 ± 74.10	21.18 ± 32.76	24.26 ± 49.96
No, *n* (% in row)	39 (84.78%)	61 (74.39%)	140 (68.29%)	238 (71.47%)

1 In the subsample that reported chronic pain.

### Exploratory and confirmatory factor analyses

To test the factor structure of the Polish PAS, the total sample was randomly divided into two subsamples. The first subsample (*n* = 168) was used to conduct Bartlett’s test of sphericity (Bartlett, 1950) to confirm that the correlation matrix was not random. Additionally, the Kaiser-Meyer-Olkin (KMO) statistic for sample adequacy was assessed, with a minimum value of 0.70 required (Kaiser, 1974). Once these conditions were met, the correlation matrix was submitted to exploratory factor analysis (EFA) using Principal Factor Analysis (PFA).

Parallel analysis and a visual scree plot were employed to determine the appropriate number of factors to retain. Oblimin rotation was applied to allow for correlations between factors. *A priori* criteria for factor adequacy were defined (Watkins, 2018). Pattern coefficients ≥ 0.39 were considered salient, based on the sample size and the recommendations by Norman and Streiner (2014). Complex loadings, which were salient on more than one factor, were excluded to maintain a simple factor structure (Thurstone, 1947). Factors with at least three salient pattern coefficients and an internal consistency coefficient (Cronbach’s α) ≥ 0.70 were considered adequate.

The factor structure was subsequently tested using confirmatory factor analysis (CFA) in the second subsample (n = 169). The following fit indices were used to assess model fit: (1) the chi-square/df ratio ≤ 3 (Kline, 2023); (2) the comparative fit index (CFI) ≥ 0.95 (Hu and Bentler, 1999); (3) the root mean square error of approximation (RMSEA) ≤ 0.05 or ≤ 0.08 (Marsh et al., 2004); (4) the standardized root mean square residual (SRMR) ≤ 0.08 (Hu and Bentler, 1999).

### Internal consistency

Internal consistency of the Polish version of the PAS was assessed using Cronbach’s alpha, which is considered the most appropriate reliability measure for Likert scales. Following standard interpretation, a Cronbach’s alpha value of ≥ 0.70 was considered indicative of satisfactory internal consistency. This same criterion was applied to assess the internal consistency of the PSS-10 in the study population (Taherdoost, 2016).

### Correlations

Pearson’s linear correlation was used to assess the relationship between the PAS and PSS-10 scales. Spearman’s rank correlation was used to examine relationships between the PAS and demographic variables such as age, gender, and family status (individuals in permanent relationships versus singles).

### Multi-group analysis

Measurement equivalence was evaluated for three non-psychological variables that define subpopulations: gender, age, and family status. Models were tested to assess metric and scalar equivalence across these groups (Caldwell, 2022; Rosseel, 2012; Epskamp, 2015).

## Results

A factor structure for the Postural Awareness Scale was conducted. The results of Bartlett’s test of sphericity (Bartlett, 1950) confirmed that the correlation matrix was not random, χ^2^(66) = 849.81, *p* < 0.001. Additionally, the Kaiser-Meyer-Olkin (KMO) measure of sampling adequacy was 0.76 (Kaiser, 1974), indicating that the data were suitable for factor analysis.

Parallel analysis and a visual inspection of the scree plot suggested a three-factor solution. However, given prior research indicating a two-factor structure, both three-factor and two-factor solutions were sequentially examined.

The three-factor solution was deemed inadequate. Although five items saliently loaded onto the third factor, three of these demonstrated complex loadings on other factors. Furthermore, the internal consistency of the third factor was unacceptably low, α = 0.33 (95% CI: 0.16–0.48).

Subsequently, the two-factor solution was explored. All pattern coefficients loaded saliently onto one factor, except for PAS item 7, which exhibited salient loadings on both factors and was therefore removed to maintain a simple structure. After removing item 7, the two-factor solution was re-evaluated.

- Factor 1: Ease/Familiarity with postural awareness. This factor was composed of five items, accounting for 24% of the variance. Its internal consistency was α = 0.82 (95% CI: 0.78–0.86).
- Factor 2: Need for attention regulation with postural awareness. This factor consisted of six items, also accounting for 24% of the variance. Its internal consistency was α = 0.80 (95% CI: 0.75–0.85).

Overall, the two-factor solution with 11 items provided the most reliable and interpretable representation of the PAS in this sample. Detailed factor loadings and item distributions are presented in Table 2.

**Table 2:** Factor structure of the Polish PAS.

Items	Factor 1: Ease/Familiarity with postural awareness	Factor 2: Need for attention regulation with postural awareness
1. I need to concentrate very much in order to become aware of my body posture.^a^ Muszę się bardzo skoncentrować, żeby uświadomić sobie, jaką mam postawę ciała.^a^	−0.09	**0.52**
2. When I assume a poor body posture, I often do not notice it until I develop pain.^a^ Kiedy przyjmuję niewłaściwą postawę ciała, często nie zauważam tego, dopóki nie pojawi się ból.^a^	0.05	**0.76**
3. When sitting, I often slump without being aware of it.^a^ Podczas siedzenia często się garbię, nie zdając sobie z tego sprawy.^a^	0.12	**0.80**
4. When I am concentrating on a specific activity, I often assume a certain body posture without knowing it.^a^ Kiedy skupiam się na konkretnej czynności, często przyjmuję określoną postawę ciała, nie wiedząc o tym.^a^	−0.04	**0.72**
5. It is difficult for me to consciously assume a specific body posture. Trudno jest mi świadomie przyjąć określoną postawę ciała.^a^	−0.12	**0.57**
6. While I am working, I regularly check my body posture. Podczas pracy regularnie sprawdzam swoją postawę ciała.	**0.73**	−0.07
7. Through my body posture, I can actively influence the impression I make on other people.^b^ Poprzez postawę ciała mogę aktywnie wpływać na wrażenie, jakie robię na innych ludziach.^b^	**-**	-
8. Throughout the day, I am continually aware of how I am currently sitting or standing. Przez cały dzień jestem stale świadoma/−y tego, jak aktualnie siedzę lub stoję.	**0.84**	0.04
9. I often call into my awareness how I am currently sitting or standing. Często uświadamiam sobie to, jak aktualnie siedzę lub stoję.	**0.73**	−0.08
10. Even during focused work, I am continually aware of my body posture. Nawet podczas pracy, która wymaga skupienia cały czas jestem świadomy swojej postawy ciała.	**0.78**	0.14
11. Through my body posture, I can consciously control my mood. Poprzez postawę ciała mogę świadomie kontrolować swój nastrój.	**0.49**	−0.21
12. I notice whether or not my body posture is good for me only when I concentrate on it.^a^ Zauważam, czy moja postawa ciała jest dobra, czy nie, tylko wtedy, gdy się na niej koncentruję.^a^	−0.13	**0.51**

Bold values indicate salient loadings.

a Reversed scoring.

b Item excluded from the final instrument.

**Figure 2:**
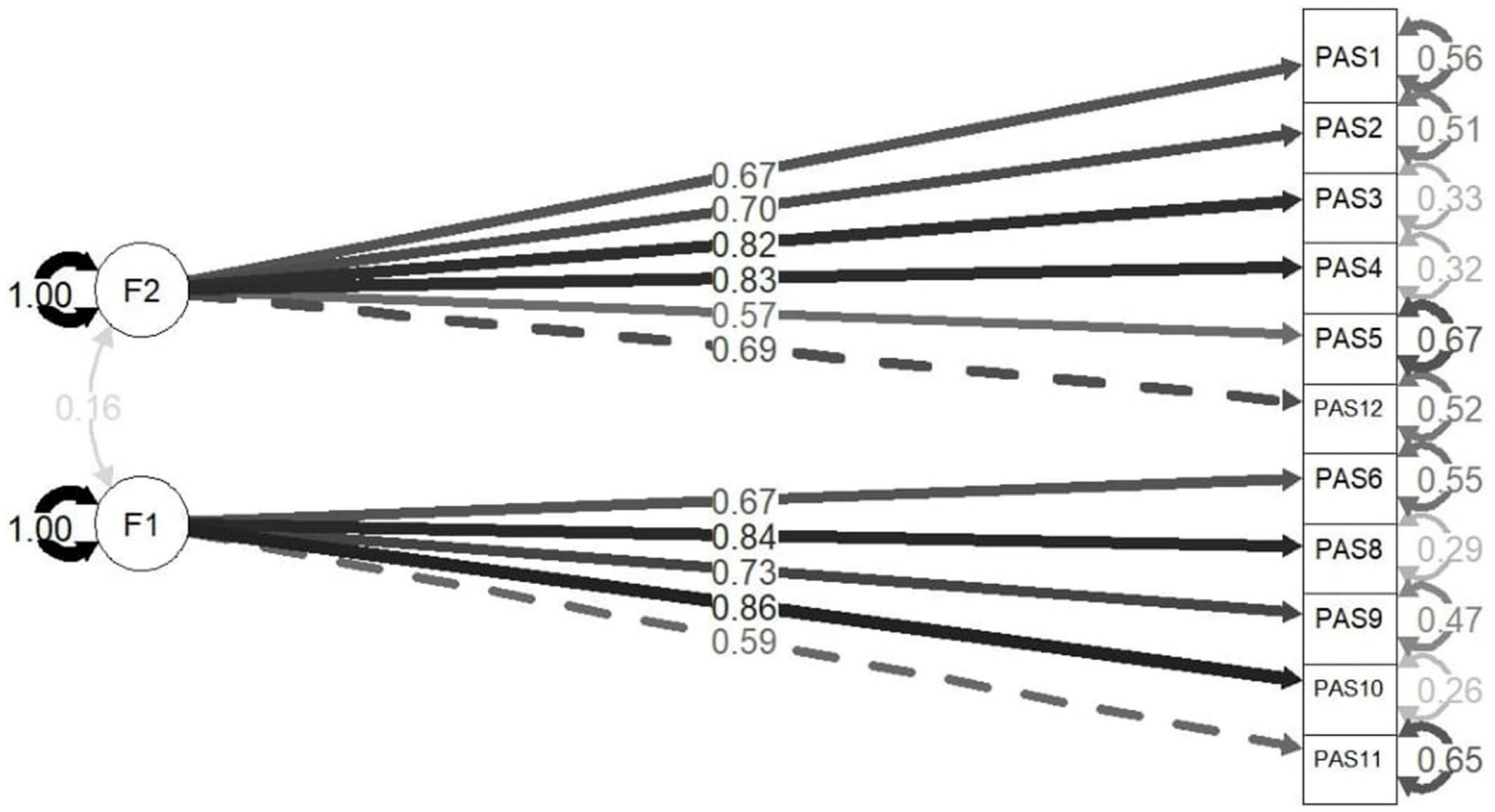
Standardized estimations.

Results of the CFA supported the two-factor solution with following model fit indicators: χ^2^/df = 1.78, CFI = 0.958, RMSEA = 0.068 (90% CI = 0.043–0.093), SRMR = 0.060. Items converged on the scales as predicted with significant standard loadings (Figure 2). The two latent factors were not significantly correlated (*p* = 0.067).

The internal consistency of the PAS (11 items) and PSS-10 questionnaires was satisfactory. For PAS, Cronbach’s alpha was 0.76, with a standardized alpha of 0.76. Split-half reliability was 0.83 for the first half and 0.82 for the second half. For PSS-10, Cronbach’s alpha was 0.82, the standardized alpha was 0.81, and split-half reliability was 0.69 for the first half and 0.67 for the second half.

Pearson correlation analysis showed significant but weak negative correlations between scores on both PAS subscales and the total PAS score and self-assessed stress level (total PSS-10 score). The negative correlation indicates a tendency to associate stronger stress levels with lower postural awareness. No significant correlations were found between body awareness and gender or family status (steady relationship or single). However, a weak but statistically significant correlation was observed between PAS Factor 1 and age. The average PAS Factor 1 score was 18.18 ± 7.34 for the group of respondents aged 61 and older, 16.05 ± 7.05 for those aged 18–35, and 16.62 ± 7.44 for those aged 36–60, indicating a trend toward greater ease and familiarity with body awareness in older people (Table 3).

**Table 3:** Relationships between PAS questionnaire scores and observed variables.

Pair of Variables	*R*	*p*
PAS Factor 1 & PSS-10 total	−0.22^a^	<0.001^*^
PAS Factor 2 & PSS-10 total	−0.13^a^	0.019^*^
PAS total score & PSS-10 total	−0.23^a^	<0.001^*^
PAS Factor 1 & Gender	0.05^b^	0.336
PAS Factor 2 & Gender	0.01^b^	0.921
PAS total score & Gender	0.01^b^	0.773
PAS Factor 1 & Family status	0.09	0.088
PAS Factor 2 & Family status	0.04	0.374
PAS total score & Family status	0.09	0.081
PAS Factor 1 & Age category	0.12^b^	0.028^*^
PAS Factor 2 & Age category	−0.07	0.158
PAS total score & Age category	0.03	0.533

* Statistically significant correlation.

a Pearson correlation.

b Spearman Rank Order Correlation.

The multigroup analysis was tested using the 11-item version of the scale. The metric equivalence test indicated no statistically significant differences in individual factor loadings or the entire vector across gender, age, and family status.

The scalar invariance test demonstrated:

- Gender: Scalar equivalence was confirmed for most loadings, except for two (PAS-7, PAS-9).
- Family Status: Scalar equivalence was confirmed for most loadings, except for one (PAS-8).
- Age: Scalar equivalence was not met for PAS 2, PAS 4, PAS 8, and for the entire scale.

These results suggest that while the PAS demonstrated strong metric and partial scalar invariance across gender and family status, scalar invariance across age groups could not be fully established (Table 4).

**Table 4:** Test of equality of factor loadings and intercepts (scalar equivalence) – non-invariant items.

Grouping variable	Item	X^2^	df	*p*
Gender	PAS 7	7.095	1	0.008
	PAS 9	6.924	1	0.009
Family status	PAS 8	6.19132	1	0.013
Age	PAS 2 (18–35 years & >60 years)	5.135	1	0.023
	PAS 2 (36–60 years & >60 years)	3.863	1	0.049
	PAS 4 (18–35 years & >60 years)	16.694	1	<0.001
	PAS 8 (18–35 years & >60 years)	23.954	1	<0.001
	Total	80.325	44	<0.001

## Discussion

This study aimed to validate the Polish version of the self-reported Postural Awareness Scale (PAS; Cramer et al., 2018). This relatively new tool assesses the ease and awareness of controlling body posture in everyday situations. The PAS questionnaire has already been validated and culturally adapted into several languages, facilitating future cross-cultural research to explore how language, worldview, and upbringing influence postural hygiene. Tools such as PAS can serve as a common instrument for interdisciplinary research teams, including psychologists, physiotherapists, physicians, dieticians, and personal trainers. Research conducted by such teams could play a crucial role in raising awareness about the importance of proper body posture for holistic psychophysical well-being.

The original German version of the PAS consists of 12 items, evenly divided into two subscales: *Ease and Familiarity with Postural Awareness* and *Need for Attention Regulation with Postural Awareness*. This two-factor structure has also been maintained in the English (Colgan et al., 2021), Italian (Topino et al., 2020), and French (Da Costa Silva et al., 2022) versions. However, the cultural adaptation of PAS into Turkish, conducted among office workers (mean age = 39.05 ± 8.44 years, primarily married men with higher education), required the removal of item 12: *Needs to concentrate to feel whether a posture benefits her/him or not* (Dursun and Önen, 2024). Similarly, the Polish version excluded item 7: *Influences her/his appeal by posture*, as it violated construct validity, confirmed by both exploratory and confirmatory factor analyses. The confirmatory factor analysis demonstrated significantly improved goodness-of-fit indices after the removal of this item. The 11-item Polish version of PAS showed satisfactory reliability and internal consistency.

Body awareness is fundamentally shaped by cultural practices, socialization, and linguistic frameworks that direct attention to specific bodily sensations and experiences (Abdoli et al., 2024). The need to remove item 7 from the Polish version reflects specific cultural nuances in how body posture is conceptualized and experienced in Polish society. Polish cultural attitudes toward body presentation tend to emphasize functionality and health over aesthetic appeal, particularly in relation to posture. This differs from Western European contexts where body posture might be more explicitly linked to social attractiveness and self-presentation (Guo et al., 2023; Hofstede, 2001).

Recent research indicates that only 20.2% of Polish adults engage in regular physical activity exceeding 30 min once a week, and more than half do not practice preventive behaviors related to proper posture and back health. Additionally, the Polish healthcare approach has traditionally focused more on treatment rather than prevention, with only 35% of Polish individuals using lumbar support during sedentary activities and less than half employing ergonomic standards for sleeping arrangements (Kuśmierek et al., 2024). This relatively low engagement with physical wellness practices suggests that many Polish individuals may have limited experiential awareness of how posture influences social perception, making item 7 less relevant to their lived experience.

The creators of the PAS have emphasized the relationship between postural awareness and perceived pain, noting that programs aimed at increasing body awareness and mindfulness can help reduce pain and depression (Cramer et al., 2018). Similarly, Jossy and Oberoi (2021) found that IT industry workers with lower postural awareness, as measured by PAS, experienced more severe neck pain. Gard (2005) reported that body awareness training can improve the quality of life in individuals with fibromyalgia. In our study, we did not divide respondents within the studied age groups depending on the occurrence of chronic pain, which may be considered a limitation, particularly in the context of the lack of scalar invariance for PAS across age groups. It is plausible that participants in the oldest age group (over 60 years) experienced chronic pain more frequently, potentially influencing the weight they assigned to individual items on the scale. However, the metric invariance assumption allowed us to estimate correlations between age and PAS scores, which revealed that postural awareness increases with age.

In contrast, Topino et al. (2020), who validated the Italian version of PAS, found no significant correlation between age and PAS scores. However, their study population had a different age structure, ranging from 18 to 77 years, with a mean age of 29. Nearly 49% of their sample were students, while only 1% were retirees. Neither our study nor those conducted in Italy (Topino et al., 2020) or France (Da Costa Silva et al., 2022) found significant correlations between PAS scores and gender. However, both the French and Italian studies reported that engagement in physical activity was associated with greater postural awareness. This positive association between physical activity and body awareness, measured by the Body Awareness Questionnaire (BAQ), has also been documented by Kalkışım et al. (2023).

No convergent and divergent validity analyses could be conducted, except for the comparison with the PSS-10 due to the lack of Polish-validated scales assessing body awareness. For this reason, the PAS was correlated only with the PSS-10 (Perceived Stress Scale). Our analysis revealed a weak but significant negative correlation, indicating that individuals experiencing higher levels of chronic stress reported lower postural awareness. These findings are consistent with previous studies suggesting that body awareness training can reduce chronic stress and enhance well-being (Mustafa and Gulati, 2022; Hepburn et al., 2021).

The study was limited by the lack of equality in the compared gender and age groups, as well as the lack of information on the type, intensity, and frequency of physical activity undertaken by respondents. These factors should be supplemented in further studies.

In light of the available literature and the findings of this study, further research on the potential utility of proprioceptive and interoceptive body awareness training in postural re-education and the treatment of chronic musculoskeletal disorders appears valuable. The rapid advancement of cognitive neuroscience presents an opportunity to evaluate the effectiveness of therapies integrating physiotherapeutic techniques with psychotherapy. Such approaches should recognize that proper, efficient, and aesthetically pleasing movement—characteristic of healthy individuals—results from the interplay between the mechanical musculoskeletal system and the learning processes in the central nervous system, which are closely linked to emotional states (Gyllensten et al., 2010; Tuthill and Azim, 2018).

## Conclusion

This study provided a reliable and validated tool for assessing postural awareness in the dimensions of *familiarity* and *attention regulation*. While the Polish version of the Postural Awareness Scale (PAS) required the removal of one item to ensure construct validity, its psychometric properties are comparable to those of the original version.

The scale is straightforward to administer and has the potential to be widely used in both research and practice. Its simplicity and clarity make it a valuable instrument for exploring the relationship between postural quality and body awareness within the body–mind model. This can have applications across various populations, including patients undergoing rehabilitation, athletes aiming to optimize performance, and general populations engaged in preventive health practices.

The availability of a Polish version of PAS opens avenues for further research into the cultural, psychological, and physiological factors influencing postural awareness. For example, it can be used to study the effects of body awareness training on stress reduction, pain management, and overall well-being. Additionally, the scale offers opportunities for interdisciplinary research, allowing psychologists, physiotherapists, and other health professionals to collaboratively investigate how body posture awareness impacts both physical and mental health outcomes.

Future studies should focus on expanding its application in clinical and non-clinical settings, as well as on examining its utility in designing interventions aimed at improving postural health and enhancing proprioceptive and interoceptive awareness. Such research could contribute to a deeper understanding of the role of postural awareness in promoting holistic well-being and addressing chronic musculoskeletal conditions.

## Data Availability

The raw data supporting the conclusions of this article will be made available by the authors, without undue reservation.

## Footnotes

^1^ https://www.oxfordreference.com/display/10.1093/acref/9780198568506.001.0001/acref-9780198568506-e-947
